# Mitochondrial Genetic Variation in Iranian Infertile Men with Varicocele

**DOI:** 10.22074/ijfs.2016.5047

**Published:** 2016-09-05

**Authors:** Mohammad Mehdi Heidari, Mehri Khatami, Amirhossein Danafar, Tahere Dianat, Ghazaleh Farahmand, Ali Reza Talebi

**Affiliations:** 1 Department of Biology, Faculty of Science, Yazd University, Yazd, Iran; 2 Department of Biology, Ashkezar Islamic Azad University, Ashkezar, Yazd, Iran; 3 Department of Biology, Faculty of Science, Islamic Azad University Shahrekord, Shahrekord, Iran; 4 Research and Clinical Center for Infertility and Department of Anatomy, Shahid Sadughi University of Medical Sciences, Yazd, Iran

**Keywords:** Infertility, Varicocele, Mutation, Mitochondrial Genes

## Abstract

**Background:**

Several recent studies have shown that mitochondrial DNA mutations lead to major disabilities and premature death in carriers. More than 150 mutations in human mitochondrial DNA (mtDNA) genes have been associated with a wide spectrum of disorders. Varicocele, one of the causes of infertility in men wherein abnormal inflexion and distension of veins of the pampiniform plexus is observed within spermatic cord, can increase reactive oxygen species (ROS) production in semen and cause oxidative stress and sperm dysfunction in patients. Given that mitochondria are the source of ROS production in cells, the aim of this study was to scan nine mitochondrial genes (*MT-COX2, MT-tRNA^Lys^ , MT-ATP8, MT-ATP6, MT-COX3, MT-tRNA^Gly^ , MT-ND3, MT-tRNA^Arg^* and *MT-ND4L*) for mutations in infertile patients with varicocele.

**Materials and Methods:**

In this cross-sectional study, polymerase chain reaction-single strand conformation polymorphism (PCR-SSCP) and DNA sequencing were used to detect and identify point mutations respectively in 9 mitochondrial genes in 72 infertile men with varicocele and 159 fertile men. In brief, the samples showing altered electrophoretic patterns of DNA in the SSCP gel were sent for DNA sequencing to identify the exact nucleotide variation.

**Results:**

Ten type nucleotide variants were detected exclusively in mitochondrial DNA of infertile men. These include six novel nucleotide changes and four variants previously reported for other disorders.

**Conclusion:**

Mutations in mitochondrial genes may affect respiratory complexes in combination with environmental risk factors. Therefore these nucleotide variants probably lead to impaired ATP synthesis and mitochondrial function ultimately interfering with sperm motility and infertility.

## Introduction

The major concern among married couples when they are unsuccessful to conceive after one year of regular unprotected intercourse is that they may be infertile. Male factors can be attributed to half of these cases (1,2). The most common surgically reversible cause of infertility is varicocele. Its prevalence is about 4.4-22.6% in the general population, 21-41% in men with primary infertility and 75-80% in men with secondary infertility (3,4). Despite the advances in molecular medicine, the pathophysiology of varicocele induced infertility remains unknown. Several proposed mechanisms include venous pressure changes and increased testicular temperature due to dilation and tortuosity of the pampiniform plexus of veins, oxidative stress, retrograde flow of renal or adrenal products, Leydig cell dysfunction and hyperthermia (5,6). In addition, a number of patients with varicocele have genetic abnormalities like Yq-microdeletions (7). Among them, oxidative stress-induced DNA damage appears to be a more likely cause which may severely affect sperm quality leading to infertility (8). This damage is one of the potential etiological factors in varicocele. A major source of partially reduced derivatives of molecular oxygen (O_2_) is mitochondria (9). The variety of reactive oxygen species (ROS) that mitochondria produce principally include hydrogen peroxide (H_2_O_2_), superoxide anion (O_2_^.-^) and the hydroxyl radical (OH^.^) (10,11). In normal physiology, ROS perform several roles in regulating cellular functions by interacting with cellular components (12). In fertile men, physiological levels of ROS play important roles in sperm function, acrosome reaction, capacitation, hyper-activation and the penetration of oocyte by spermatozoa. However, in varicocele patients ROS generation is abnormally enhanced (13,14). 

Specific point mutations and deletions of mitochondrial DNA (mtDNA) have been associated with poor sperm motility and semen quality in several studies. Sperm mtDNA is highly sensitive to mutations due to increased ROS by-products generated during oxidative respiration (15). When large amounts of mutant mtDNA accumulate in the testes, reduction in ATP production, mitochondrial respiratory dysfunction and meiotic arrest are induced in spermatogonia cells (16). Each mitochondrion has 2-10 mitochondrial genomes responsible for coding the subunits of the OXPHOS complex. The OXPHOS machinery is made up of over 80 different polypeptides, of which the mtDNA encodes 13 polypeptides including complex I, III, IV and V subunits (17). 

In this study, for first time, we further analyzed nine genes (*MT-COX2, MT-tRNA^Lys^, MT-ATP8, MT-ATP6, MT-COX3, MT-tRNA^Gly^, MT-ND3, MT-tRNA^Arg^*and *MT-ND4L*) in the mitochondrial genome by polymerase chain reaction-single strand conformation polymorphism (PCR-SSCP) assay and direct sequencing techniques to identify the possible association between mtDNA variation with varicocele in the Iranian population.

## Materials and Methods

### Patients

This study was a cross sectional study. Seventy two Iranian infertile men with clinical varicocele were recruited in the study. The varicocele diagnosis was made by the urologists for the patients by physical examination in standing position and via scrotal palpation in a temperature controlled room (23°C). Semen analysis was performed according to the WHO laboratory manual (18). Patients with varicocele were in 3 grades: i. Grade I (n=12), ii. Grade II (n=27) and iii. Grade III (n=33). The control group (healthy volunteers) consisted of 159 fertile and normospermic men from the Yazd Infertility Center who fathered at least one child. The ethnic and geographical origin of all patients and controls was the same. All participants were fully informed of the objectives of the study and those that signed the consent form were assigned to the study. All infertile men in the age group ranging from 22 to 36 years (mean, 29 years) were referred for evaluation of their infertility (1 year of unprotected intercourse and not leading to conception). The Yazd University Ethics Committee approved recruitment of patients and laboratory protocols in this study. 

### DNA extraction and mutation analysis

Peripheral blood samples were obtained from varicocele patients and the DNA was extracted using a standard salting-out procedure. Purified DNA samples from leukocytes were used for the PCR reactions. To amplify *MT-COX2, MT-tRNA^Lys^ , MT-ATP8, MT-ATP6, MT-COX3, MT-tRNA^Gly^ , MT-ND3, MT-tRNA^Arg^* and *MT-ND4L* mitochondrial genes, four pairs of PCR primers were designed, which were located in the flanking regions of each gene (Table 1). Primer Design was based on the human mitochondrial sequence by primer design software (Primer Premier 5.0; Premier Biosoft Inc., Canada), and their secondary structure was examined with Gene Runner version 3.05 (Hastings Software Inc. Hastings, NY, USA, http://www.generunner.com). Each reaction was prepared to a final volume of 25 µl containing 1XMasterMix PCR (Yekta Tajhiz Azma Co., Iran), 0.2 mM of each primer and 0.5 µg DNA template. The PCR conditions were an initial denaturation of 95°C for 5 minutes followed by 35 cycles of denaturation at 95°C for 30 seconds, the annealing temperature (Table 1) for 30 seconds and extension at 72°C for 30 seconds, which was extended for 5 minutes in the final cycle. The PCR products were electrophoresed on an ethidium bromide-stained 2% agarose gel.

**Table 1:** Primers used for mitochondrial genes


Segment	Primersequence(5'-3')	Primer position	Tm(ºC)	Size(bp)	Gene

Seg.1	F:CTACGGTCAATGCTCTGAAA	8161-8180	56.5	309	*MT-COX2, MT-tRNA^Lys^, MT-ATP8, MT-ATP6*
	R: TAGGTGGTAGTTTGTGTTTA	84708451			
Seg.2	F:AGCCCACTTCTTACCACAAG	8901-8920	56	338	*MT-ATP6*
	R: TACTATATGATAGGCATGTGA	9239-9219			
Seg.3	F:CACTATCTGCTTCATCCGCC	9851-9870	57		*MT-COX3, MT-ND3*
	R: ATGTAGCCGTTGAGTTGTGG	10150-10131			
Seg.4	F:TCTGGCCTATGAGTGACTAC	10361-10380	57	221	*MT-ND4L, MT-tRNA^Arg^*
	R: AGTATTATTCCTTCTAGGCA	10582-10380			


Tm; Temperature melting.

For the SSCP assay, PCR products were heatdenatured at 95 ºC for 5 minutes and chilled on ice for 5 minutes, and then loaded onto an 8% nondenaturing polyacrylamide/TBE 0.5x gel. Gels were stained with silver nitrate to reveal the bands of single strand DNA. Various band patterns of the amplified PCR products were marked and scored. The typical gene variants got sequenced using a commercial company (Macrogen, South Korea). All the data obtained from automated sequencing was checked with Sequencher. The online multiple sequence alignment software ClustalW2 (http:// www.ebi.ac.uk/tools/msa/clustalw2/) and BLAST analysis were used to determine the nature of mutations and percent homology of the sequences that have been obtained in the study with all other sequences of five other species (chimpanzee, monkey, cattle, zebrafish and drosophila). 

### Software and databases

We used the tool PolyPhen-2 (http://genetics.bwh.harvard.edu/pph2/) for prediction of the functional consequences of mutations and damaging effect of missense mutations on protein structure. The sequence alignment was performed using the blastp program available at the National Center for Biotechnology Information (NCBI) web site (http://www.ncbi.nlm.nih.gov/Blastp) and the ClustalW program (http://bioinfo.hku.hk/services/ analyseq/cgi-bin/clustalw_in.pl). For detection of structural features of mammalian mitochondrial tRNAs and human diseases linked to point mutations in mitochondrial tRNA genes, we used Mamit-tRNA (http://mamit-trna.u-strasbg.fr). 

### Statistical analysis

The GraphPad Prism software (GraphPad Software, Inc. USA) was used for statistical analysis. Distributions of continuous variables in groups were expressed as mean ± SD, and compared with unpaired Student’s t test. P<0.05 were regarded as statistically significant. 

### Results

**Fig.1:**
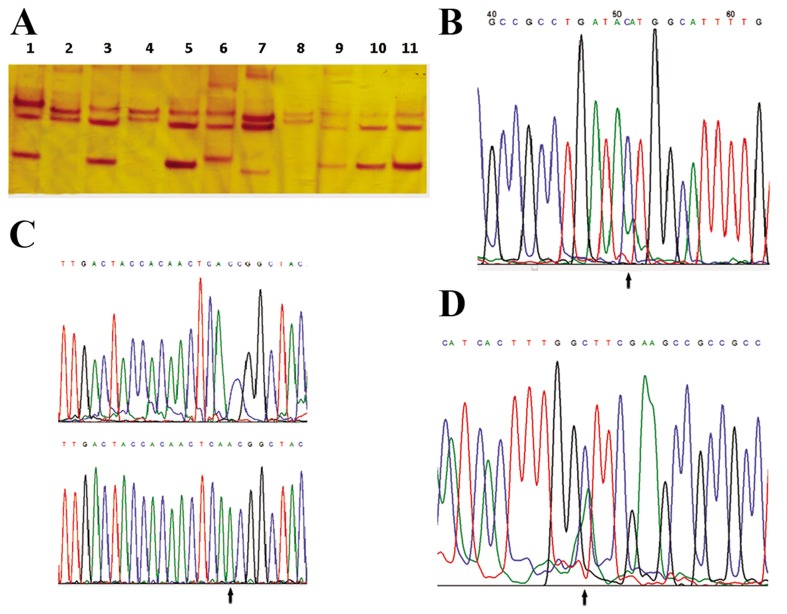
Silver staining SSCP analysis of fragment 3. A. Polyacrylamide gel electrophoresis. Lanes 1, 3 and 5 show 3 patients who did not have mutations, B. Lane 6 shows a patient with the m.9929 C>A mutation, C. Lanes 2, 4 and 8 show 2 patients with the m.10141A>C mutation, and, D. Lane 7 shows a patient with m.9911C>A and lanes 9, 10 and 11 are men without varicocele.

The age difference between the 72 Iranian infertile men with varicocele (mean age of 30.76 ± 6.47) and 159 normal controls (mean age: 28.8 ± 6.01) was not significant (P=0.785). Mutation analyses for the mitochondrial *MT-COX2, MTtRNA^Lys^, MT-ATP8, MT-ATP6, MT-COX3, MTtRNA^Gly^, MT-ND3, MT-tRNA^Arg^* and *MT-ND4L* genes were carried out in all of patients and healthy controls by PCR-SSCP. Mobility shift of single strand DNA on polyacrylamide gel electrophoresis was the criterion for sequencing and the identification of DNA variation (Fig .1). We found ten different nucleotide substitutions of which 4 caused an amino acid change, of which one occurred in tRNA^Arg^. None of the ten mutations were found in healthy controls. All the mutations identified are summarized in Table 2. In addition, 6 were novel mutations of which four were silent mutations. Four reported polymorphisms, including m.8258T>C, m.9911C>A, m.9932G>A and m.10463T>C were found in six patients. The m.9911C>A variant in *MT-COX3* was heteroplasmic. The novel 9 bp heteroplasmic insertion was found in the non-coding MT-NC7 locus in one patient. 

**Fig.2:**
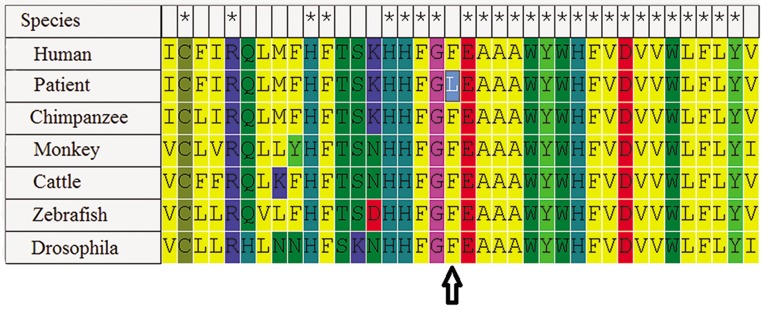
Protein alignment of m.9911C>A missense mutation *MT-COX3* and the arrow indicate the site of the F235L mutation.

The m.9911C>A mutation, an aromatic amino acid phenylalanine codon (TTC), changes to leucine codon (TTA), a hydrophobic amino acid at position 235 (designated F235L) in 1 patient (Fig .2) and the novel 9929C>A mutation changes a polar tyrosine to threonine. 

Two novel mutations were detected in 5 patients with one (9929C>A) being a nonsense mutation and changes tyrosine to stop codon (Y241X) and the other (10141C>G) being a missense mutation that changes Asparagine to Lysine (N27K). Also, three synonymous polymorphisms were found that were not reported previously (Table 2). The other variation was the m.10463T>C substitution (homoplasmic state) in the tRNA^Arg^gene that was found in 3 patients. 

**Table 2:** Mitochondrial variation found in infertile men with varicocele


Locus	Position	Nucleotide change	Amino acid position	No. of individuals	Hetero/Homo	Previously reported

MT-COX2	8258	T→C	F225L	1	Homo	Yes (19)
MT-NC7	Ins8288	9 bp	Non-coding	1	Hetero	No
MT-COX3	9911	C→A	F235L	1	Hetero	Yes (20)
MT-COX3	9929	C→A	Y241X	2	Hetero	No
MT-COX3	9932	G→A	W242W	1	Homo	Yes (21, 22)
MT-ATP6	9063	A→G	L179L	1	Homo	No
MT-ND3	10103	A→G	L15L	1	Homo	No
MT-ND3	10141	C→A	N27K	3	Homo	No
MT-TR	10463	T→C	tRNA^Arg^	6	Homo	Yes (23, 24)
MT-ND4L	10550	A→G	M27M	12	Homo	No


## Discussion

One of the most frequent causes of male infertility is varicocele, however, the pathogenic mechanisms by which it leads to changes in spermatogenesis are not clear (25). Some of these mechanisms may be related to mutations in mitochondrial complexes that affect flagellar movement and cause sperm dysmotility. 

DNA alterations including point mutations and deletions of mtDNA have been reported in infertile patients with low sperm motility (26). The effect of mtDNA mutations on male infertility has also been studied. Shamsi et al. (27) reported that generation of ROS and mtDNA mutations are associated with pathogenic molecular mechanisms. Agarwal et al. (28) showed an increased oxidative stress in varicocele patients. Thangaraj et al. (29) demonstrated that sperm mitochondrial mutations is one of the causes of low sperm motility which is strongly dependent on ATP biosynthesis which is carried out by the mitochondrial OXPHOS system. Furthermore, it has been demonstrated that cells with some base substitutions in mtDNA can greatly influence semen quality (9,30,31). 

It has been established that mitochondrial dysfunction caused by mtDNA mutations and oxidative damages is one of the important reasons for most types of infertility such as Varicocele (32). The mtDNAs alterations may accumulate in the spermatids or during gametogenesis and thereby impair the respiratory function and motility of spermatozoa (33). 

We observed three heteroplasmic variations in 4 patients. A nine base pair heteroplasmic insertion in the non-coding MT-NC7 locus were found in 1 patient. Although this insertion (5ˊ-CCCCCTCTA-3ˊ) has been found in a noncoding region, it may cause mitochondrial rearrangements and DNA strand break affected by topoisomerases or DNA recombinase (34). 

The heteroplasmic m.9911C>A and m.9929C>A transversions in *MT-COX3* alter two conserved codons. Given that these variants change highly conserved amino acids and were not identified in normal controls, they may be considered as pathogenic mutations for the following reasons. First, these missense mutations are found in several patients. Second, these mutations are not reported as polymorphisms in the general population and are not detected in the control individuals from the same ethnic background. Third, the mutations are heteroplasmic in the lymphocyte cells. Fourth, we propose that these mutations may affect the polarity of the protein due to the replacement of a natural amino acid with a polar amino acid. Using PolyPhen2, we found that these mutations are expected to change protein function. 

Here, we describe seven homoplasmic variants in 25 patients: two missense mutations in *MT-COX2* (8258T>C) and *MT-ND3* (10141C>A), 4 synonymous polymorphisms in *MT-COX3, MTATPase6, MT-ND3* and *MT-ND4L* and one mutation (10463T>C) in *MT-tRNA^Arg^*. This mutation is located at a moderate conserved region of the acceptor stem of tRNA arginine. This mutation was not observed in healthy control subjects but was previously reported as a polymorphism in mitochondrial encephalomyopathy (35) and may be one of the several predisposing factors for varicocele. 

## Conclusion

Because sperms require an optimal energy to reach the oviduct during fertilization, the appropriate bioenergetic function of mitochondria is critical for male infertility. Therefore, any changes in mitochondrial genome can cause improper functioning of respiratory chain that in combination with environmental risk factors lead to infertility in men. This first Iranian study revealed that some Iranian infertile men carry variants in the nine mitochondrial genes and suggests that variants in these genes may be associated with varicocele. 
